# A lucky catch: Fishhook injury of the tongue

**DOI:** 10.4103/0974-2700.58653

**Published:** 2010

**Authors:** Karen A Eley, Daljit K Dhariwal

**Affiliations:** Department of Oral & Maxillofacial Surgery, Oxford Radcliffe Hospitals NHS Trust, Oxford, UK

**Keywords:** Fishhook, tongue, airway

## Abstract

Fishhook injuries, particularly those involving the upper limbs, are frequently encountered in recreational and commercial fishing settings. The oral cavity is rarely a site for such injury. We present the case of a 13-month-old male child who sustained a fishhook injury to the tongue whilst ‘playing’ with an unused fishhook at home. In this case there was minimal swelling, and the fishhook could be uneventfully removed under general anesthesia. Penetrating injuries to the tongue carry the risk of swelling and hematoma formation, which may result in airway compromise. These injuries therfore call for early intervention.

## INTRODUCTION

The majority of injuries caused by fishhooks are penetrating soft tissue injuries to the upper limbs and are encountered in recreational and commercial fishing settings.[1] One-third of such injuries affect the face, and the literature is replete with management guidance for injuries involving the orbit.[2–4] The oral cavity appears to be a rare site for such injury, with only one reported case of a soft palate injury in a child.[5] To the best of our knowledge, no injuries involving the tongue have previously been reported in the literature.

## CASE HISTORY

**Figure 1:**
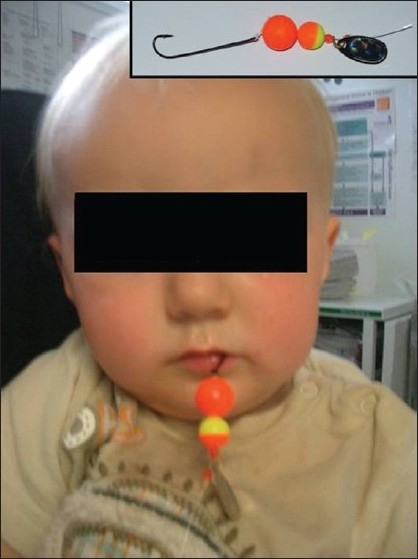
Fishhook embedded within tongue in child reluctant to open mouth and inset, the offending hook

A 13-month-old male presented to the emergency department with a fishhook embedded in his tongue and was immediately referred to the maxillofacial team. The boy had found a brightly colored unused fishing hook whilst playing at home and had placed it in his mouth [Figure 1]. When he attempted to pull the hook out from his mouth, it had become embedded in the dorsal aspect of his tongue. Attempts to remove the hook by his parents had resulted in it becoming further fixed within the tissues. There was no airway compromise, and his vital signs were within normal limits. He was transferred to the operating theater and the fishhook was uneventfully removed by a retrograde technique under general anesthesia. There was minimal swelling of the posterior aspect of the tongue. Antibiotics were not considered necessary. His childhood immunisations were up to date. He was discharged after a period of observation.

## DISCUSSION

Injuries to the tongue carry significant risk of haematoma and potentially rapid swelling, threatening the airway. Patients should be managed with special attention to airway protection, nursed in the upright position, and all potentially distressing interventions should be minimized. Prompt removal of the fishhook should be performed under direct vision to prevent further soft tissue damage. General anesthesia is warranted in young children who are unable to cooperate. Local anesthesia is often required for all but the most superficial injuries. There are four main techniques described for fishhook removal: retrograde, string-yank, needle-cover, and advance-and-cut.[1] Identification of the hook type will aid in selecting the most appropriate removal technique. Doser *et al.*[6] found that 40% of fishhooks could be removed with a retrograde approach, which follows the entry path of the hook and therefore minimises further soft tissue damage. Where the fishhook has multiple barbs, any barbs that are not embedded in the tissues should be protected to prevent them from becoming so. The surgeon should take care to avoid personal injury, and eye protection should be worn. The fishhook should be inspected to confirm that no fragments have been left within the wound; often the patient will be able to provide another unused fishhook for comparison.

Following removal, a period of observation is advised, with the duration depending upon the extent of soft tissue swelling. The fishhook that had caused the injury in our patient was a new unused one, which is unusual in such injuries. In any case, few injuries involving fishhooks are associated with infection, and systemic antibiotics are rarely required provided thorough wound irrigation is carried out. As with all penetrating injuries, tetanus immunization status should be checked and cover provided where appropriate.

## CONCLUSION

All penetrating injuries of the tongue require prompt assessment to ensure adequate airway protection. In an uncooperative child, this can be difficult and examination often depends upon subtle signs. Management should be anticipatory and instituted early.
